# Exploiting solvent effects in drug design and optimization

**DOI:** 10.1186/1758-2946-6-S1-P43

**Published:** 2014-03-11

**Authors:** Jean-Francois Truchon, Kristina Grabowski, Barbara Sander, Alain Ajamian

**Affiliations:** 1 Vertex, Laval, H7V 4A7, Canada; 2 Chemical Computing Group, Köln, 50672, Germany; 3 Chemical Computing Group, Montreal, H3A 2R7, Canada

## 

Upon ligand binding, solvent molecules around the binding pocket and the ligand become displaced or rearranged. These desolvation energies can be a significant portion of the total binding energy, and thus represent opportunities for ligand design. Computing desolvation energetics typically requires lengthy simulations, but this talk presents a fast and easy-to-use method (3D-RISM) which computes desolvation energies in minutes, without using explicit simulations. Application to ligand optimization is demonstrated using case studies.

## References

[B1] Luchko T Gusarov S Roe DR Simmerling C Case DA Tuszynski J Kovalenko A Three-dimensional molecular theory of solvation coupled with molecular dynamics in Amber J Chem Theory Comput 2010 6 607 624 10.1021/ct900460m 20440377 PMC2861832

[B2] Kovalenko Hirata F Self-consistent description of a metal-water interface by the Kohn-Sham density functional theory and the three-dimensional reference interaction site model J Chem Phys 1999 110 10095 10112 10.1063/1.478883

